# Excitonic Au_4_Ru_2_(PPh_3_)_2_(SC_2_H_4_Ph)_8_ cluster for light-driven dinitrogen fixation†

**DOI:** 10.1039/c9sc06424a

**Published:** 2020-01-27

**Authors:** Yongnan Sun, Wei Pei, Mingcai Xie, Shun Xu, Si Zhou, Jijun Zhao, Kang Xiao, Yan Zhu

**Affiliations:** Key Lab of Mesoscopic Chemistry, School of Chemistry and Chemical Engineering, Nanjing University Nanjing 210093 China zhuyan@nju.edu.cn; Key Laboratory of Materials Modification by Laser, Ion and Electron Beams, Dalian University of Technology Dalian 116024 China sizhou@dlut.edu.cn; School of Materials Science and Engineering, Nanjing University of Posts and Telecommunications Nanjing 210023 China

## Abstract

The surface plasmon resonance of metal nanoparticles has been widely used to improve photochemical transformations by plasmon-induced charge transfer. However, it remains elusive for the molecular-like metal clusters with non-metallic or excitonic behavior to enable light harvesting including electron/hole pair production and separation. Here we report a paradigm for solar energy conversion on an atomically precise Au_4_Ru_2_ cluster supported on TiO_2_ with oxygen vacancies, in which the electron–hole pairs can be directly generated from the excited Au_4_Ru_2_ cluster and the TiO_2_ support, and the photogenerated electrons can transfer to the Ru atoms. Importantly, the Ru atoms in the Au_4_Ru_2_ cluster are capable of injecting the electrons into adsorbed N_2_ to activate N_2_ molecules. The cooperative effect in the supported Au_4_Ru_2_ catalyst efficiently boosts the photocatalytic activity for N_2_ fixation in comparison with homogold (Au_*n*_) clusters.

## Abstract 2

A molecular-like Au_4_Ru_2_ cluster supported on TiO_2_ with oxygen vacancies enables light harvesting to drive the fixation of N_2_.

## Graphical abstract

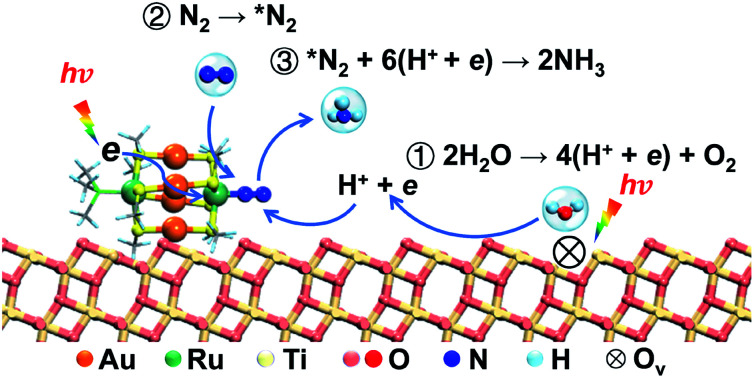

## Introduction

Atomically precise metal clusters with exact formulas, molecular purity, and total structures have gathered momentum in recent years, owing to their unique physical and chemical properties.^1–6^ The metal clusters in the quantum size regime possess discrete electron energy levels and show non-metallic or excitonic behaviours,^7^ which are totally different from the larger metallic-state nanoparticles exhibiting a distinct surface plasmon resonance.^8,9^ Significant advances in chemical synthesis of the clusters provide an exciting opportunity to unveil previously unknown or inaccessible insights into the applications in optics, catalysis, and biochemistry.^10,11^ Especially the cluster-based heterogeneous catalysts have exhibited new catalytic properties in many chemical reactions compared to the plasmonic metal nanoparticles.^12,13^ Furthermore, the precise relation of the properties with atomic-level structures not only reveals the origin of metal catalysis, but also promotes the exploration of important chemical processes with these clusters as well-defined, highly efficient catalysts.^14,15^

One important chemical process is the reduction of dinitrogen to ammonia, which is an essential chemical and energy carrier.^16^ However, high temperature and pressure are necessary to drive the reaction of N_2_ with H_2_ to NH_3_, due to the strong nonpolar triple bond in N_2_ and its high activation barrier.^17^ Many efforts have been made to develop less energy-consuming alternatives that can overcome the kinetic limitation of NH_3_ production.^18–21^ Inspired by nitrogenase enzymes that can fix nitrogen under ambient conditions, nanostructured metal catalysts are springing up to enable N_2_ fixation with the help of photosynthesis.^22–25^ Despite the important advances in homogeneous complex systems, construction of heterogeneous metal sites for N_2_ fixation is currently still challenging. Considering that atomically precise metal clusters can bridge the gap between homogeneous and heterogeneous catalysts, we speculate whether metal clusters with excitonic behaviour can convert N_2_ into ammonia under mild conditions, that is, whether these clusters are capable of generating hot electrons driven by solar light, ensuring the charge separation, and then donating electrons into N_2_. If this scenario is feasible, it can not only unravel the mystery of non-plasmon-induced solar energy conversion but also offer fundamental insights into exact, heterogeneous metal sites to govern the N_2_ transformation at the unprecedented level of atomic precision.

**Fig. 1:**
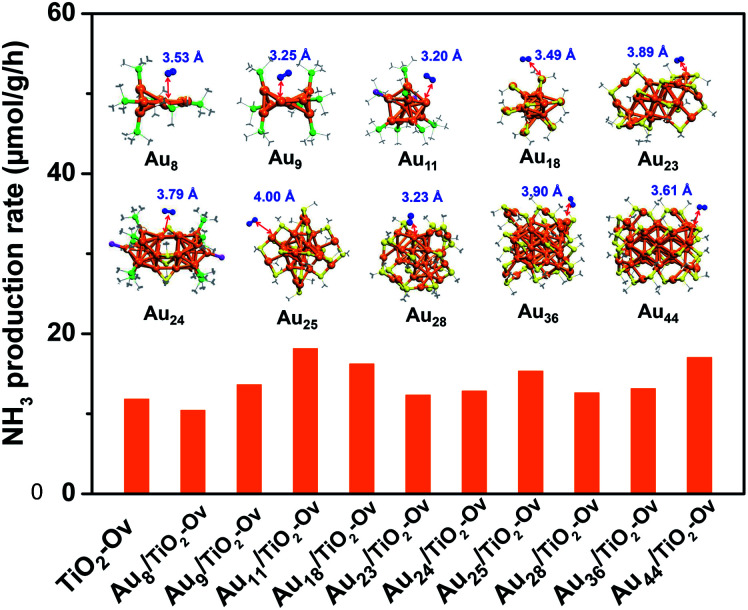
The photocatalytic activity of the ligand-protected Au_*n*_ clusters supported on TiO_2_-Ov for N_2_ reduction. The inset shows the atomic structures of the Au_*n*_ clusters, which can only physisorb N_2_ molecule with distance between N_2_ and Au sites larger than 3.2 Å. The C, N, S, Au, P and Cl are shown in gray, blue, yellow, orange, green and violet colors, respectively. H atoms are omitted for clarity.

Since no example of N_2_ conversion on atomically precise metal clusters has been documented, a series of ligand-protected Au_*n*_ (*n* = gold atom number) clusters with different atomic structures were first screened to catalyse the N_2_ conversion under light irradiation. As shown in Fig. 1, these Au_*n*_ clusters failed to give a convincing activity for photocatalytic reaction of N_2_ fixation, mainly because the N_2_ molecule cannot coordinate to clusters and be activated on the gold sites of the Au_*n*_ clusters, according to our density functional theory (DFT) calculations (inset of Fig. 1). We next turned our attention to the bimetal clusters. Since Ru is recognized as a suitable candidate for N_2_ fixation,^24^ we sought to explore the wet chemical synthesis of atomically precise Au–Ru clusters.

In this work, we successfully synthesized a new Au_4_Ru_2_ cluster protected by thiolate and triphenylphosphine ligands (namely, Au_4_Ru_2_(PPh_3_)_2_(SC_2_H_4_Ph)_8_) and solved its crystal structure. Excitingly, the Au_4_Ru_2_ cluster supported on TiO_2_ with oxygen vacancies (hereafter denoted as TiO_2_-Ov) exhibited a drastic increase in the photocatalytic activity for N_2_ reduction compared to the supported Au_*n*_ catalysts. Furthermore, we explicitly demonstrated the cooperative mechanism within the Au_4_Ru_2_/TiO_2_-Ov catalyst for achieving light-driven N_2_ fixation.

## Results and discussion

**Fig. 2:**
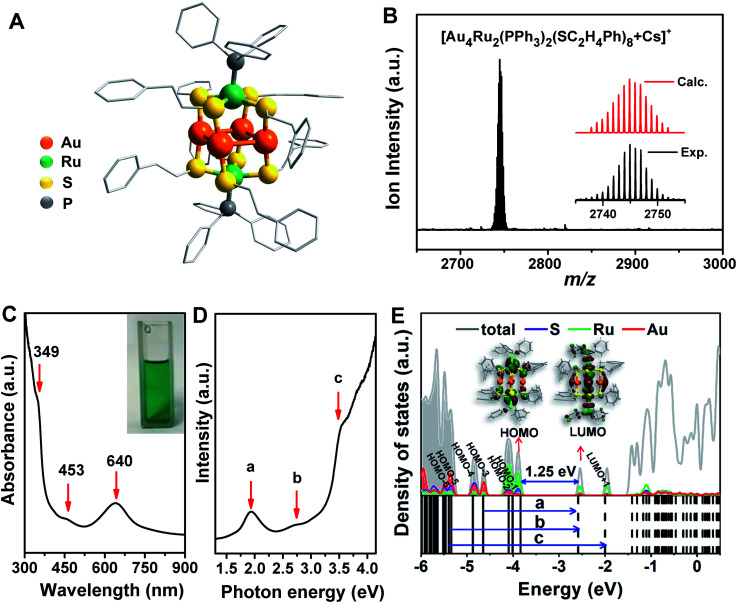
(A) Atomic structure (H atoms are omitted for clarity) and (B) ESI-MS profile of Au_4_Ru_2_(PPh_3_)_2_(SC_2_H_4_Ph)_8_. (C) UV-vis spectrum of Au_4_Ru_2_(PPh_3_)_2_(SC_2_H_4_Ph)_8_. (D) UV-vis spectrum plotted on the photon energy scale. (E) Kohn–Sham orbitals (bottom panel) and density of states (top panel) of Au_4_Ru_2_(PPh_3_)_2_(SC_2_H_4_Ph)_8_ from DFT calculations and the corresponding charge density distributions of HOMO and LUMO (insets).

The crystal structure of the Au_4_Ru_2_(PPh_3_)_2_(SC_2_H_4_Ph)_8_ cluster is shown in Fig. 2A. This cluster resembles a distorted hexahedron, in which four Au atoms are located at the midpoints of four side edges, two Ru atoms reside on the centres of the top and the bottom planes, and eight S atoms are fixed at the vertexes. The two apex Ru atoms are coordinated by two PPh_3_ with the average Ru–P bond length of 2.204 Å. Four S atoms binding to a Ru atom are within the same plane as indicated by the average S–Ru–S angle of 90°. The average S–Au–S angle is 172.9°, where S–Au bond distances are 2.310 and 2.318 Å, respectively. The Au–Au distances fall in a very narrow range of 3.045–3.144 Å, which are shorter than the sum of van der Waals radii of two Au atoms (3.32 Å), suggesting the presence of d^10^–d^10^ metallophilic contact within the Au_4_Ru_2_ cluster.^26^ Electrospray ionization mass spectrometry (ESI-MS) further confirmed the formula of the cluster, where the *m*/*z* 2745 peak was assigned to [Au_4_Ru_2_(PPh_3_)_2_(SC_2_H_4_Ph)_8_ + Cs]^+^ adduct supported by the agreement between experimental and simulated isotopic patterns (Fig. 2B). Thermogravimetric analysis further confirmed that the Au_4_Ru_2_ cluster was highly pure (Fig. S1†).

UV-vis absorption spectrum of the Au_4_Ru_2_ cluster shows two prominent peaks at 349 and 640 nm and one weak peak at 453 nm (Fig. 2C), corresponding to excitation energies of 3.55, 1.94 and 2.74 eV (Fig. 2D), respectively. Accordingly, the optical gap is determined to be 1.33 eV based on the photon-energy scale spectrum, which is basically consistent with the computed gap of 1.25 eV between the highest occupied molecular orbital (HOMO) and lowest unoccupied molecular orbital (LUMO) from our DFT calculations (Fig. 2E). As revealed by the computed electronic density of states (DOS) in Fig. 2E, the emergence of discrete electronic states and a moderate HOMO–LUMO gap for the Au_4_Ru_2_ cluster indicates single electron excitations, that is an exciton.^7^ Importantly, the projected DOS shows that the low-lying unoccupied states (LUMO and LUMO+1) are mostly localized on the Ru atoms, suggesting that the excited carriers in the Au_4_Ru_2_ cluster will be on the Ru atoms, which may act as reaction centers and utilize the excess electrons for N_2_ activation.

**Fig. 3:**
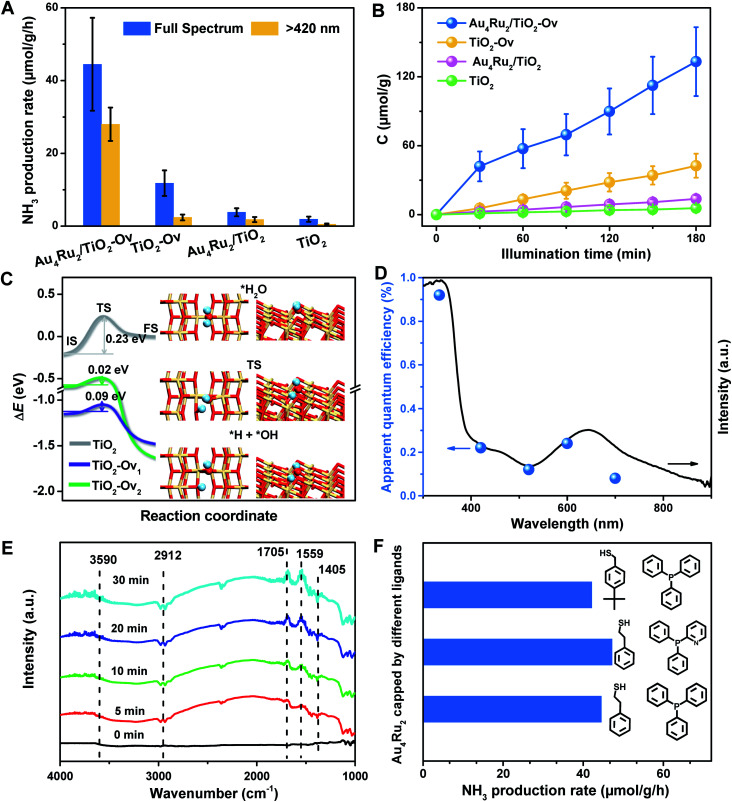
(A) The photocatalytic performances of the Au_4_Ru_2_/TiO_2_-Ov, Au_4_Ru_2_/TiO_2_, TiO_2_-Ov and TiO_2_ catalysts for N_2_ reduction. (B) Time-dependent photocatalytic ammonia production over the Au_4_Ru_2_/TiO_2_-Ov, Au_4_Ru_2_/TiO_2_, TiO_2_-Ov and TiO_2_ catalysts. (C) Reaction energy diagrams of water dissociation on the anatase TiO_2_(101) surface without defect (TiO_2_) and with an oxygen vacancy per supercell on surface (TiO_2_-Ov_1_) and subsurface (TiO_2_-Ov_2_), respectively. Δ*E* is relative to the energy of each substrate plus a free water molecule. Insets are the structures of water adsorption (top) and dissociation into *H and *OH species adsorbed on TiO_2_-Ov_1_ (bottom), and the transition state (middle). The H, O and Ti are shown in cyan, red and yellow. (D) UV-DRS and quantum efficiency of Au_4_Ru_2_/TiO_2_-Ov for NH_4_^+^ evolution by photocatalytic N_2_ reduction under monochromatic light of different wavelengths. (E) *In situ* FTIR spectra of the photocatalytic N_2_ reduction over Au_4_Ru_2_/TiO_2_-Ov during full spectrum illumination in the presence of N_2_ and water. (F) Performances of the Au_4_Ru_2_ clusters protected by different ligands for photocatalytic N_2_ reduction.

With the newly synthesized cluster, we explored the proposed light-driven N_2_ fixation using Au_4_Ru_2_ as a heterogeneous catalyst. As shown in Fig. 3A, the Au_4_Ru_2_/TiO_2_-Ov catalyst gave rise to an ammonia production rate of 44.5 μmol g^−1^ h^−1^ under full spectrum illumination, which exhibited over 3-fold increase in photocatalytic activity compared to the Au_*n*_/TiO_2_-Ov and pure TiO_2_-Ov catalysts. As much, the Au_4_Ru_2_/TiO_2_-Ov catalyst resulted in a 4-time higher activity than TiO_2_-Ov in visible light-driven N_2_ reduction (Fig. 3A), suggesting a strong synergistic effect between Au_4_Ru_2_ and TiO_2_-Ov. Time-dependent photocatalytic ammonia production over the Au_4_Ru_2_ catalysts revealed that, not only the ammonia concentration increased linearly with the irradiation time in the visible light region (Fig. 3B), but also the Au_4_Ru_2_ loaded on the TiO_2_ support without abundant oxygen vacancies gave a much lower activity driven by either UV-vis or visible light (Fig. 3A and B). In fact, both TiO_2_-Ov and TiO_2_ substrates were in anatase phase (Fig. S2A†). The difference in the two TiO_2_ samples was that the former contained oxygen vacancies, but the latter not, which was confirmed by electron paramagnetic resonance (EPR). TiO_2_-Ov showed a characteristic EPR signal at approximately *g* = 1.998, suggesting the presence of oxygen vacancies,^27^ whereas no EPR signal was observed on the other TiO_2_ sample (Fig. S2B†). It can be conjectured that the abundant oxygen vacancies in TiO_2_ facilitate the photochemical reaction of N_2_ reduction.^17^

DFT calculations demonstrated that TiO_2_ with oxygen vacancies can efficiently promote the photolysis of water to produce hydrogen as the proton source of ammonia (Fig. S3†). The anatase TiO_2_(101) surfaces with an oxygen vacancy on the surface (TiO_2_-Ov_1_) and subsurface (TiO_2_-Ov_2_), have low kinetic barriers (Δ*E*_a_) of 0.09 and 0.02 eV for water dissociation, respectively, compared to 0.23 eV for the perfect TiO_2_(101) surface (Fig. 3C). Moreover, they all provide moderate binding strength with H atoms (binding energy Δ*E*_H_ = 0.14–0.43 eV relative to the energy of H_2_ molecule), which is beneficial for protons transfer from TiO_2_ to Au_4_Ru_2_. Photocatalytic N_2_ fixation on the Au_4_Ru_2_/TiO_2_-Ov catalyst in CH_3_CN solvent did not produce ammonia, again corroborating the origin of protons in ammonia from water splitting. For comparison, the Au_4_Ru_2_ cluster supported on SiO_2_ gave a low ammonia production rate of 2.4 μmol g^−1^ h^−1^, implying the key role of TiO_2_-Ov in water splitting.

Furthermore, the action spectrum for NH_3_ formation on the Au_4_Ru_2_/TiO_2_-Ov catalyst was determined under monochromatic light irradiation at wavelengths of 334, 420, 520, 600, and 700 nm. The trend of apparent quantum efficiencies (AQEs) well matched that of the optical absorption spectrum of the Au_4_Ru/TiO_2_-Ov (Fig. 3D). This proved that the photocatalytic N_2_ fixation originated from the light absorption by the Au_4_Ru_2_ cluster. In addition, the catalytic activity decreased slightly with multiple cycles (Fig. S4†), mainly due to the partial detachment of Au_4_Ru_2_ from TiO_2_-Ov (∼8 wt% metal loss detected by inductively coupled plasma-atomic emission spectroscopy (ICP-AES) analysis). The diffuse reflectance optical spectra (DRS) of the Au_4_Ru_2_/TiO_2_-Ov sample did not significantly change after the reaction (Fig. S5A and B†) and transmission electron microscopy (TEM) studies showed that the spent catalyst had no obvious aggregation (Fig. S5C and D†), suggesting that the Au_4_Ru_2_ cluster was robust throughout the reaction.

To directly visualize the N_2_ conversion on the Au_4_Ru_2_/TiO_2_-Ov catalyst, *in situ* infrared Fourier transform (DRIFT) spectroscopy was utilized to monitor the time-dependent change of the functional nitrogenous intermediates on the surface of Au_4_Ru_2_/TiO_2_-Ov. No signal change was observed in the DRIFT spectra within 30 min of incident light exposure in the absence of water (Fig. S6†), suggesting that the H atoms in ammonia indeed came from water. After water was introduced into the reaction cell, several absorption peaks appeared gradually with the irradiation time (Fig. 3E). The broad band at 3590 cm^−1^ is assigned to the *ν*(N–H) stretching vibration, and the two absorption bands at 1705 and 1559 cm^−1^ are attributed to the *σ*(N–H) bending vibration.^25,28^ Besides, the bands at 1405 and 2912 cm^−1^ assigned to the NH_4_^+^ deforming vibration became stronger gradually with the irradiation time.^29,30^ The result validated that the Au_4_Ru_2_/TiO_2_-Ov catalyst can convert N_2_ into ammonia under the light irradiation.

Considering that the Au_4_Ru_2_ cluster contained thiolate and PPh_3_ ligands, it is natural to ask whether the ligands can affect the catalytic conversion of N_2_. To address this, the comparison experiments were conducted, where a series of Au_4_Ru_2_ clusters protected by different ligands were prepared (Fig. S7†). As shown in Fig. 3F, the Au_4_Ru_2_ clusters with different ligands showed no drastic difference in the photocatalytic performance for N_2_ reduction, manifesting that the catalytic reaction was mainly determined by metal sites, rather than the ligands. Furthermore, when all the ligands in Au_4_Ru_2_ were removed *via* the thermal treatment, the Au_4_Ru_2_ clusters crashed into large nanoparticles and hence lost the activity for N_2_ fixation (Fig. S8†).

**Fig. 4:**
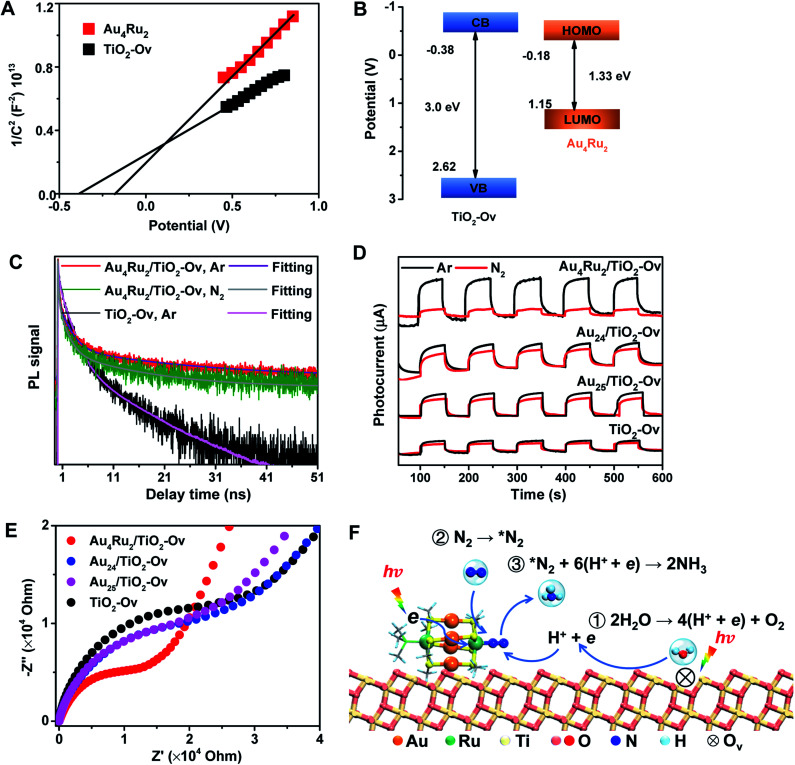
(A) Mott–Schottky plots of Au_4_Ru_2_ and TiO_2_-Ov (potential V *vs.* Ag/AgCl at pH 6.4). (B) Schematic diagram of band alignment (potential V *vs.* Ag/AgCl at pH 6.4). VB means valence band, and CB denotes conduction band. (C) Time-resolved PL spectra of the Au_4_Ru_2_/TiO_2_-Ov and TiO_2_-Ov under Ar and N_2_ atmospheres, respectively. (D) Photocurrent responses of Au_4_Ru_2_/TiO_2_-Ov, Au_24_/TiO_2_-Ov, Au_25_/TiO_2_-Ov and TiO_2_-Ov under Ar and N_2_ atmospheres, respectively. (E) Electrochemical impedance spectra of Au_4_Ru_2/_TiO_2_-Ov, Au_24_/TiO_2_-Ov, Au_25_/TiO_2_-Ov and TiO_2_-Ov in the presence of 300 W xenon lamp. (F) Proposed mechanism for the photocatalytic N_2_ reduction on the Au_4_Ru_2_/TiO_2_-Ov catalyst.

We now discuss the mechanism that Au_4_Ru_2_/TiO_2_-Ov can achieve an extraordinary activity for photocatalytic N_2_ reduction, but Au_*n*_/TiO_2_-Ov and TiO_2_-Ov cannot. Mott–Schottky (M–S) plots were first collected to provide the flat band potentials of Au_4_Ru_2_ and TiO_2_-Ov. The obtained tangent positive slopes indicated that both Au_4_Ru_2_ and TiO_2_-Ov were likely n-type semiconductors (Fig. 4A). The flat band potentials of Au_4_Ru_2_ and TiO_2_-Ov *versus* the saturated Ag/AgCl were −0.18 and −0.38 V, respectively. Based on the UV-DRS and M–S measurements, the band alignments of Au_4_Ru_2_ and TiO_2_-Ov were shown in Fig. 4B. From the thermodynamic point of view, photogenerated electron carriers can transfer from TiO_2_-Ov to Au_4_Ru_2_, while the Au_4_Ru_2_ cluster was more capable of light-driven N_2_ reduction than the TiO_2_-Ov substrate.

The charge carrier kinetics of the Au_4_Ru_2_ cluster with N_2_, including separation, transfer and recombination, was investigated by room temperature steady-state and time-resolved photoluminescence (PL) spectroscopy. As shown in Fig. S9,† when the Ar atmosphere was changed to the N_2_ atmosphere, the steady-state PL spectrum of Au_4_Ru_2_/TiO_2_-Ov was significantly quenched, which was related to the non-radiative transfer of the photoexcited electrons from Au_4_Ru_2_ to the π* antibonding orbitals of N_2_ adsorbed on the cluster.^30^ The time-resolved PL spectroscopy studies (Fig. 4C) showed that the average decay time (*τ* = 14.40 ns) of Au_4_Ru_2_/TiO_2_-Ov was longer than that of TiO_2_-Ov (*τ* = 5.26 ns). The prolonged lifetime of the photogenerated electrons illustrated that Au_4_Ru_2_ supported on TiO_2_-Ov can reduce the charge recombination, thereby possess highly effective separation of electron–hole pairs.^31^

We argued that the high efficiency of N_2_ fixation on the Au_4_Ru_2_/TiO_2_-Ov catalyst was acquired by a synergic effect between Au_4_Ru_2_ and TiO_2_-Ov. To confirm this, transient photocurrent responses were conducted on the Au_4_Ru_2_/TiO_2_-Ov, TiO_2_-Ov, and two reference systems (Au_24_/TiO_2_-Ov and Au_25_/TiO_2_-Ov) under the Ar and N_2_ atmospheres with light (Fig. 4D), respectively. Compared to TiO_2_-Ov, the photocurrents of Au_24_/TiO_2_-Ov and Au_25_/TiO_2_-Ov were enhanced under the Ar atmosphere, suggesting that the Au_*n*_ clusters also can serve as trapping sites for the photogenerated electrons.^30^ However, the transient photocurrent responses of TiO_2_-Ov, Au_24_/TiO_2_ and Au_25_/TiO_2_-Ov samples under N_2_ atmosphere were similar to those under Ar atmosphere, indicating that the interfacial electron transfer in the three samples was not interfered by surrounding N_2_. It partially accounted for the catalytic performance of the Au_*n*_/TiO_2_-Ov catalysts shown in Fig. 1 that the Au_*n*_/TiO_2_-Ov catalysts did not significantly enhance the photoactivity of N_2_ reduction in comparison with the TiO_2_-Ov. Notably, the photocurrent of the Au_4_Ru_2_/TiO_2_-Ov sample under the N_2_ atmosphere was only a quarter of that under the Ar atmosphere (Fig. 4D), in which the quenching of the other three-quarters of photocurrent was possibly due to the electrons consumed by the adsorbed N_2_ molecules.

Moreover, electrochemical impedance spectroscopy (EIS) was measured to investigate the interfacial charge-transfer properties of the above four samples under illumination. As shown in Fig. 4E, the semicircular diameters of Au_24_/TiO_2_-Ov and Au_25_/TiO_2_-Ov measured under light irradiation were slightly smaller than that of TiO_2_-Ov, indicating that the Au_*n*_ clusters had an inherent ability of electron transport, but this ability was not extraordinary. Notably, the impedance of Au_4_Ru_2_/TiO_2_-Ov was the smallest among the four samples, providing a solid evidence that there existed a fast transfer of the interfacial charges between Au_4_Ru_2_ and TiO_2_-Ov.^32^ The charge transfer resistance on the Au_4_Ru_2_/TiO_2_-Ov sample without illumination was also investigated (Fig. S10†). It was found that the impedance of Au_4_Ru_2_/TiO_2_-Ov in the absence of light was much higher than that in the presence of light. Therefore, these observations supplied a clue that the Au_*n*_ clusters were able to generate the electrons under the light irradiation, but lacked the ability to activate N_2_, and thus the Ru atoms in Au_4_Ru_2_ should be crucial for N_2_ binding and activation. To further elucidate the critical contribution of the Ru atoms in the Au_4_Ru_2_ cluster to the activation of the inert N≡N triple bond, the two Ru atoms of Au_4_Ru_2_ were replaced by the two Pd atoms (Fig. S11A†), that is, Au_4_Pd_2_. No increase in the photocatalytic reduction of N_2_ on the Au_4_Pd_2_/TiO_2_-Ov was observed when compared to the Au_*n*_/TiO_2_-Ov (Fig. S11B†). The result definitely confirmed that the Ru atoms in the Au_4_Ru_2_ cluster indeed can provide unique reaction sites for the N≡N cleavage by strong coordination.

**Fig. 5:**
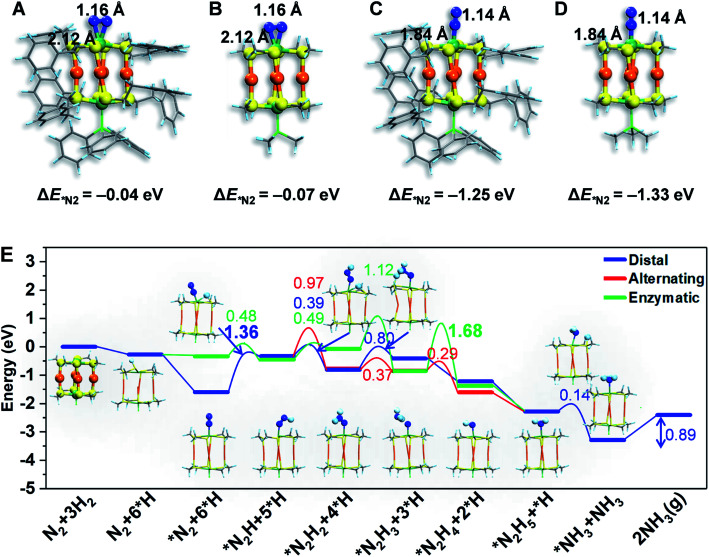
The atomic structures of N_2_ molecule adsorption *via* (A and B) end-on and (C and D) side-on patterns on the Au_4_Ru_2_(SC_2_H_4_Ph)_8_PPh_3_ and Au_4_Ru_2_(SCH_3_)_8_P(CH_3_)_3_. The H, C, N, S, Au and Ru are shown in light blue, gray, blue, yellow, orange and blackish green colors, respectively. The adsorption energy of a N_2_ molecule is shown for each system, revealing that simplifying the ligands of –PPh_3_ and –SC_2_H_4_Ph by –P(CH_3_)_3_ and –SCH_3_, respectively, has a minor effect on the binding properties of the cluster with N_2_. (E) The colors numbers indicate the kinetic barriers of N_2_ fixation on the Au_4_Ru_2_ cluster, and the barriers of rate-limiting step are bolded. The atomic structures of reaction intermediates are displayed as insets. The H, C, N, S, Au and Ru are shown in light blue, gray, blue, yellow, orange and green colors, respectively.

To gain atomistic insight into the photochemical N_2_ reduction on the Au_4_Ru_2_/TiO_2_-Ov catalyst, we performed DFT calculations to determine the active sites and reaction pathways. Our calculations show that the Au atoms do not have any activity for N_2_ fixation, but N_2_ can be adsorbed onto the Ru atom in the side-on or end-on configuration, with adsorption energies of −0.07 eV and −1.33 eV and Ru–N bond length of 2.14 Å and 1.84 Å, respectively (Fig. 5A–D). The N–N bond is elongated to 1.14–1.16 Å compared to 1.13 Å for the gaseous N_2_ molecule, which manifests that N_2_ is activated on the Ru site of the Au_4_Ru_2_ cluster. Moreover, we examined the structure of Au_4_Ru_2_(SCH_3_)_8_(P(CH_3_)_3_)_2_ cluster supported on the anatase TiO_2_(101) surface (Fig. S12†), which exhibits a weak interfacial interaction with a distance of 2.57 Å between the cluster and substrate and a binding energy of only −0.28 eV per Au(Ru) atom. Therefore, the presence of substrate should not affect the adsorption properties of the Au_4_Ru_2_ cluster with N_2_. Hereafter, we considered N_2_-to-NH_3_ conversion on the Au_4_Ru_2_ cluster without support of the substrate.

Ammonia synthesis on the Au_4_Ru_2_ cluster can proceed through three pathways, *i.e.* distal, alternating and enzymatic mechanisms (Fig. 5E).^24,33^ For the former two paths, the N_2_ molecule strongly binds with the underlying Ru atom in the end-on configuration. Protonation of the chemisorbed *N_2_ species to form a *NNH intermediate is endothermic and has a kinetic barrier of 1.36 eV, which is the rate-limiting step of N_2_-to-NH_3_ conversion. The following reaction steps are exothermic involving barriers below 0.97 eV or even barrierless. For the enzymatic mechanism, the adsorption strength of *N_2_ species on Ru is relatively weak. The reaction proceeds almost down-hill in energy. Reaction from *N_2_ to form a *HN–NH species is favorable with a kinetic barrier of only 0.48 eV. Protonation of *H_2_N–NH leads to the breaking of N–N bond and generation of two *H_2_N species, which requires the largest barrier of 1.68 eV during the whole reaction. Finally, desorption of *NH_3_ has to overcome a moderate energy barrier of 0.89 eV. In brief, our DFT calculations suggest that the synergistic effect of the Au_4_Ru_2_/TiO_2_-Ov catalyst stems from the cooperation between cluster and substrate during the catalytic reaction (Fig. 4F): the Ru atom in the Au_4_Ru_2_ cluster serves as the active site for N_2_ fixation and ammonia synthesis through the distal or alternating pathways; the anatase TiO_2_(101) substrate plays important roles in water splitting to generate hydrogen protons that transfer to the cluster for the N_2_-to-NH_3_ reaction.

## Conclusions

In conclusion, we have synthesized an excitonic Au_4_Ru_2_ cluster, which enables light harvesting including electron/hole pair production and separation. The experimental studies combined with theoretical calculations demonstrate that the cooperative effect between Au_4_Ru_2_ cluster and TiO_2_ substrate with oxygen vacancies leads to an extraordinary activity for light-driven N_2_ reduction. The electron–hole pairs can be generated from the excited Au_4_Ru_2_ cluster; the heterojunction between Au_4_Ru_2_ cluster and the TiO_2_-Ov substrate also facilitates photocarriers separation; the photoelectrons transfer to the Ru atoms of the cluster; meanwhile, TiO_2_-Ov induces water splitting to produce hydrogen protons for N_2_ fixation and conversion on the Ru atoms. Certainly, this work provides deep insights into non-plasmon-induced charge transfer from atomically precise metal clusters and develops a feasible strategy to enable highly efficient solar energy utilization *via* pursuing heterogeneous catalysts with atomic precision.

## Conflicts of interest

No conflicts of interest.

## Supplementary Material

SC-011-C9SC06424A-s001

SC-011-C9SC06424A-s002
