# Supplementary material for: Excitonic Au4Ru2(PPh3)2(SC2H4Ph)8 cluster for light-driven dinitrogen fixation
Source: Chem Sci. 2020 Jan 27;11(9):2440–7. doi: 10.1039/c9sc06424a (PMC8157179; doi:10.1039/c9sc06424a)
Supplement: SC-011-C9SC06424A-s001 [file SC-011-C9SC06424A-s001.pdf]

Electronic Supplementary Material (ESI) for Chemical Science.
This journal is © The Royal Society of Chemistry 2020

Supporting Information

Excitonic $Au_4Ru_2(PPh_3)_2(SC_2H_4Ph)_8$ cluster for light-driven dinitrogen fixation

Yongnan Sun,[1,#] Wei Pei,[2,#] Mingcai Xie,[1] Shun Xu,[1] Si Zhou,*[,2] Jijun Zhao,[2] Kang Xiao,[3] and Yan Zhu*[,1]

[1]Key Lab of Mesoscopic Chemistry, School of Chemistry and Chemical Engineering, Nanjing University, Nanjing 210093, China

[2]Key Laboratory of Materials Modification by Laser, Ion and Electron Beams, Dalian University of Technology, Dalian 116024, China

[3]School of Materials Science and Engineering, Nanjing University of Posts and Telecommunications, Nanjing 210023, China

Experimental Section

Materials

Tetrachloroauric (III) acid ($HAuCl_4 \cdot 4H_2O$, >99.9% metals basis, Aldrich), Triphenylphosphine gold(I) chloride (98%, Aldrich), Dichloro(pentamethylcyclopentadienyl) ruthenium(III) polymer (98%, MERYER), Triphenylphosphine ruthenium chloride (99%, Aldrich), 2-Phenylethanethiol (98%, Acros Organics), Triphenylphosphine (PPh_3, 98%, Aldrich), Tetraoctylammonium bromide (TOAB, ≥98%, Aldrich), 4-tert-butylbenzenethiol (TBBT, 97%, J&K), Cyclohexanethiol (SC_6H_{11}, 98%, Aldrich), Sodium borohydride ($NaBH_4$, Aldrich), Methanol (AR, ≥99 %, Sinopharm Chemical Reagent Co., Ltd), Ethanol (AR, ≥99 %, Sinopharm Chemical Reagent Co., Ltd), Toluene (AR, >99%, Sinopharm Chemical Reagent Co., Ltd), dichloromethane (AR, >99%, Sinopharm Chemical Reagent Co., Ltd), Tetrahydrofuran (AR, >99%, Sinopharm Chemical Reagent Co., Ltd), Acetonitrile (AR, >99%, Sinopharm Chemical Reagent Co., Ltd), Titanium(IV) butoxide (97%, Aldrich), Hydrofluoric acid (HF, Sinopharm Chemical Reagent Co., Ltd), Titanium(IV) oxide (P25, 25 nm primary particle size (TEM), 98%, Macklin)

Synthesis of $Au_4Ru_2(PPh_3)_2(SC_2H_4Ph)_8$: 0.14 mmol $AuPPh_3Cl$ and 0.055 mmol $Ru(PPh)_3Cl_2$ were dissolved in 6 mL ethanol. After vigorously stirring for 15 min, 6 mL ethanol solution of 14 mg $NaBH_4$ was added to the above solution. After 2 h, the mixture was evaporated. The product was separated using 15 mL CH_2Cl_2 and 140 μL 2-phenylethylthiol was then added to give a dark solid. Then, the mixture was kept at 50 ^oC for 2 h. The crude product was separated from the reaction mixture by washing with n-

hexane and extraction with CH_2Cl_2. The crude product dissolved in CH_2Cl_2 was pipetted on PTLC plate (10 cm × 20 cm), and the separation was conducted in developing tank (developing solvent: CH_2Cl_2) for 20 min. A knife was used to cut the bands in the PTLC plate, and the green product was extracted by pure CH_2Cl_2. Green needle-like crystals of $Au_4Ru_2(PPh_3)_2(SC_2H_4Ph)_8$ were crystallized from CH_2Cl_2/menthol for 3~5 days.

Synthesis of $[Au_9(PPh_3)_8](NO_3)_3$:[S1] $NaBH_4$ (1.2 mg) dissolved in ethanol (2.5 mL) was added into $Au(PPh_3)(NO_3)$ (50 mg). After 2 h of stirring at room temperature, the reaction mixture was evaporated and then dissolved in dichloromethane (5 mL). After centrifugation and evaporation, the obtained solid was washed with THF. Finally, green powder was obtained.

Synthesis of $[Au_8(PPh_3)_7](NO_3)_2$:[S2] The $[Au_8(PPh_3)_7](NO_3)_2$ clusters were synthesized by reacting $[Au_9(PPh_3)_8](NO_3)_3$ clusters with excess PPh_3 (380 mg).

Synthesis of $[Au_{11}(PPh_3)_8Cl_2]$:[S3] 100 mg $Au(PPh_3)Cl$ was dissolved in 5 mL dichloromethane. 1.9 mg $NaBH_4$ dissolved in 2 mL ethanol was added to this above solution under stirring at room temperature for 24 h, and then the solvent was evaporated. The residue was dissolved in a minimum amount of CH_2Cl_2 and the product was precipitated with 20 times this volume of pentane. The resulting mixture was centrifuged, and the supernatant was discarded. The residue was stirred with pentane (20 mL) and centrifuged. The final solid was dissolved in EtOH, transferred to a round-bottom flask, and the solvent was evaporated to obtain the crude product.

Synthesis of $Au_{18}(SC_6H_{11})_{16}$:[S4] 0.2 mmol $HAuCl_4{\cdot}4H_2O$ dissolved in 0.5 mL ethanol was added in 10 mL CH_2Cl_2 solution of 0.232 mol TOAB under vigorously stirring for 15 min. 125 µL 1-cyclohexanethiol was added in the mixture at room temperature. After 30 min, 20 mg $NaBH_4$ of 2 mL ethanol solution was added to the above solution. The mixed solution was stirred for 5 h. Finally, the obtained product washed with methanol and extracted with CH_2Cl_2.

Synthesis of $Au_{24}(PPh_3)_{10}(SC_2H_4Ph)_5Cl_2$:[S5] 0.23 mmol $HAuCl_4{\cdot}4H_2O$ was first dissolved in 5 mL water, and then 180 mg PPh_3 was added into organic phase under vigorous stirring. 26 mg $NaBH_4$ of 5 mL ethanol solution was added to the above solution. After vigorous stirring for 4 h, the reaction product was separated using 10~12 mL CH_2Cl_2, and 200 µL 2-phenylethylthiol was added to the solution. Then, the mixture was kept at 40 ^oC for 5~8 h. Then, 1.2 g PPh_3 was added in the above mixture, and the reaction was continued for 24 h at 40 ^oC. Finally, the product washed with n-hexane and extracted with toluene.

Synthesis of $Au_{25}(SC_2H_4Ph)_{18}$:[S6] 0.203 mmol of $HAuCl_4{\cdot}3H_2O$ and 0.235 mmol of TOAB were dissolved in 15 mL tetrahydrofuran in an ice bath. Until the above solution turned a wine red (30min), 140 µL of phenylethanethiol was added into the above solution. The mixture was slowly stirred about 1~3 h, then 5 mL cold water of 78 mg $NaBH_4$ was added. After stirring for 3 h, the ice water bath was removed. Then, the mixture solution was stirred to proceed overnight. The obtained product was washed with methanol five times. Finally, the $Au_{25}(SC_2H_4Ph)_{18}$ clusters were extracted with CH_3CN.

Synthesis of $Au_{28}(TBBT)_{20}$:[S7] 10 mg $Au_{25}(SC_2H_4Ph)_{18}$ was added to the mixture of 0.5 mL toluene and 0.5 mL TBBT. The reaction solution was heated at 80 ^oC oil bath for 2 h.

The reaction product was washed with methanol three times. Finally, the $Au_{28}(TBBT)_{20}$ clusters were extracted with CH_2Cl_2.

Synthesis of $Au_{36}(TBBT)_{24}$ and $Au_{44}(TBBT)_{28}$:[S7,S8] The $Au_{36}(TBBT)_{24}$ and $Au_{44}(TBBT)_{28}$ clusters were synthesized by a two-step size focusing process. In the first step, $HAuCl_4·3H_2O$ (90 mg) of 2 mL water was added to 15 mL of CH_2Cl_2 solution containing TOAB (154 mg) in a 50 mL flask. After 30 min, the water phase was removed, then 125 µL TBBT was added to the organic phase. The reactants are stirred vigorously in an ice bath. After 4 h, 50 mg $NaBH_4$ dissolved in 5 mL water was quickly poured into the reaction mixture. The mixture was stirred to proceed overnight in the ice bath. The reaction product was washed with methanol three times to remove redundant TOAB and TBBT. In the second step, the product was divided evenly into two parts. One of parts is added with 0.5 mL toluene and 100 µL TBBT at 80 ^oC for 22 h, and then the $Au_{36}(TBBT)_{24}$ clusters were separated from the reaction mixture by washing with methanol and extraction with CH_2Cl_2. The other part was added 0.5 mL toluene and 100 µL TBBT and kept at 60 ^oC for 22 h, and then the $Au_{44}(TBBT)_{28}$ clusters were separated from the reaction mixture by washing with methanol and extraction with CH_2Cl_2.

Synthesis of $Au_{23}(SC_6H_{11})_{16}$:[S9] 0.3 mmol $HAuCl_4·3H_2O$ and 0.348 mmol TOAB were dissolved in 15 mL methanol. After vigorously stirring for 15 min, 1.6 mmol 1-cyclohexanethiol was added to the mixture at room temperature. After 60~120 min, $NaBH_4$ (3 mmol dissolved in 6 mL cold water) was added to the solution under vigorous stirring. The solution turned black immediately, which then precipitated out of the methanol

solution. The reaction mixture was further allowed to overnight and finally gave rise to pure $Au_{23}(SC_6H_{11})_{16}$ clusters.

Synthesis of $Au_4Pd_2(SC_2H_4Ph)_8$:[S10] 0.12 mmol $HAuCl_4{\cdot}4H_2O$, 0.38 mmol $Pd(NO_3)_2{\cdot}2H_2O$, 150 µL 2-phenylethylthiol and 150 µL triethylamine were dissolved in 6 mL acetonitrile under vigorously stirring for 5 h. The mixture was washed with water and methanol several times and extraction with CH_2Cl_2. Then, the $Au_4Pd_2(SC_2H_4Ph)_8$ clusters were separated by PTLC (CH_2Cl_2: petroleum ether, 1/3 v: v).

Synthesis of TiO_2-Ov: [S11] 10 mL tetra-butyl titanate was added to a mixture of 1.2 mL HF and 30 mL ethanol, which was stirred for 30 min. The above solution was transferred to a 100 mL hydrothermal kettle and kept at 180 ^oC for 2 h. After 2 h, the product was filtered and washed with water, and then dried at 60 ^oC for 24 h.

Synthesis of TiO_2: Commercially purchased titanium(IV) oxide (P25, 25 nm primary particle size (TEM), 98%, Macklin) was calcined at 400 ^oC for 4 h to obtain TiO_2.

Preparation of supported Au_4Ru_2 catalysts: 10 mg $Au_4Ru_2(PPh)_3(SC_2H_4Ph)_8$ was dissolved in 2 mL CH_2Cl_2. Then 500 mg TiO_2-Ov was added into the above solution under vigorous stirring for 10 h. Finally, the Au_4Ru_2/TiO_2-Ov catalyst was obtained by removing the solvent in flowing nitrogen at room temperature. The actual loadings of Au and Ru on the Au_4Tu_2/TiO_2-Ov catalyst were 0.56 wt% and 0.14 wt% respectively by ICP-AES analysis. The preparation methods of other supported cluster catalysts were the same as the above described method.

Characterization

The X-ray crystallography was performed on a Bruker D8 VENTURE with Mo Kα radiation (λ = 0.71073 Å). The crystal structures were resolved by direct methods and refined by full-matrix least-squares methods with SHELXL-2013 (Sheldrick, 2013). UV/vis/NIR spectra with a range of 300-1400 nm were recorded on a UV3700 spectrophotometer (SHIMADZU). Diffuse reflectance UV-vis spectra were recorded on a UV-vis spectrometer using SHIMADZU UV-3600. Electrospray ionization (ESI) mass spectra were collected on a Waters Q-TOF mass spectrometer using a Z-spray source. The sample was first dissolved in toluene (0.5 mg/mL) and then diluted (2:1 v/v) with a methanol solution containing 50 mM CsOAc. The morphology and size of catalysts were characterized using a JEOL JEM-2100F field-emission high-resolution transmission electron microscope operated at 200 kV. The XRD patterns were recorded with an X'Pert PRO MPD (PANalytical) diffractometer using Cu Kα as the radiation source at 40 kV and 40 mA. Electron paramagnetic resonance (EPR) spectra were conducted on a Bruker 500 spectrometer at room temperature. The loading weight of metal was determined via the inductively coupled plasma–atomic emission spectroscopy (ICP-AES, Optima 5300DV).

A home-built wide-field fluorescence microscope based on Olympus IX73 was used with 450 nm CW diode laser for the fluorescence spectra measurements and 450 nm pulse laser from super continuous laser (Fianium SC-400) for the PL lifetime measurement. The fluorescence was collected by a dry objective lens (Olympus LUCPlanFI 40×, NA=0.6) and detected by an EMCCD camera (iXon Ultra 888, Andor). A transmission grating (Newport, 150 lines/mm) was put in front of the camera for the spectra measurement. The

PL lifetime measurement was done by using a single photon counting system (TCSPC, Picoharp 300).

Thermal gravimetric analyses (TGA) were performed using a NETZSCH Instruments TGA STA-449C under N_2 flow. Samples were run from 20 to 800 °C with a heating rate of 10 °C/min.

In situ diffuse reflectance FTIR spectra were collected using Nicolet iS50FT-IR spectrometer (Thermo, USA) with a designed reaction cell. The substrate (Au_4Ru_2/TiO_2-Ov film) is lying at the center of the reaction cell. Then, the gas in the reaction cell and the gas adsorbed on the surface of the catalyst was extracted by an ultra-high vacuum pump. A layer of water molecules was necessary to provide protons in our functional model. Therefore, the water vapor in the nitrogen gas is passed through the substrate to form a layer of adsorbed water molecules, after which the molecular N_2 is pumped into the cells to obtain a N_2 atmosphere. Finally, the Xe lamp was turned on and the in situ FTIR spectra was collected using a MCT detector along with the reaction.

The photoelectrochemical (PEC), electrochemical impedance spectroscopy (EIS) measurement and Mott-Schottky plots were performed on a CHI660B electrochemical workstation in a standard three-electrode system with a platinum foil as a counter electrode, a glassy carbon as working electrode and a saturated Ag/AgCl as a reference electrode. A 300W Xe lamp was utilized as the light source on the PEC and EIS measurement. PEC and ESI measurements were carried out at room temperature in 0.5 mol/L Na_2SO_4, which had been deoxygenated by bubbling high-purity Ar/N_2 for 30 min. The Nyquist plots were recorded from 1 to 100 kHz frequency range at an applied 0.5 V bias voltage.

Apparent Quantum Efficiency Calculation:

Apparent quantum efficiency (AQE) is defined as the following equation.[S12]

Φ_{AQY} (%) = 100 × ([NH_3 formed (mol)] × 3)/ [photon number entered the reaction vessel (mol)]

Catalytic reaction: Photocatalytic N_2 reduction performance was tested using a quartz glass as the photocatalytic reactor under atmospheric pressure and ambient temperature, and a 300W Xe lamp (CEL-HXF300) was used as a light source. Specific catalytic evaluation scheme was as follows: 100 mg catalyst (0.7 wt% metal from ICP), 20 mL water and a magnetic stir bar were loaded into the quartz glass. The reactor was equipped with a circulating water shell to maintain room temperature. The suspension was stirred constantly in the dark, and a constant high-purity N_2 (>99.9%) at a flow rate of 50 mL/min for 30 min was bubbled to obtain a saturated aqueous suspension of N_2. The mixture was irradiated afterward with a 300W Xe lamp. 3 mL of the reaction solution was taken out every 30 min by a syringe, and then the catalyst was removed by centrifugation immediately. Concentration of NH_4^+ was determined using Nessler's reagent method[S11] on a SHIMADZU UV-1800 spectrometer and ion chromatography (930 compact IC Flex), which the and standard curves for NH_4^+ with Nessler's reagent and ion chromatography were shown in Figure S13. The content of N_2H_4 was determined using colorimetry of para-(dimethylamino) benzaldehyde.[S13] No NH_4^+ was detected when bubbling Ar only, indicating that background NH_3 was not produced in the system. In addition, the photocatalytic activity for H_2 as byproduct production was investigated and the rate of H_2 production was 3.45 μmol/g/h on the Au_4Ru_2/TiO_2-Ov under full spectrum illumination.

However, given the established fact that H_2 production in N_2 photofixation is not a serious issue, NH_3 highly dissolves in water and is easily separated from gaseous H_2. Therefore, in this study, we focused on the evolution of ammonia.

The detailed preparation method of the standard curve for $NH_4^+$of Nessler's reagent was as follows. In alkaline environment, ammonia can react with mercury iodide and potassium iodide to produce a reddish gelatinous precipitate, which has an absorption peak at 420 nm. The specific method to obtain the standard curve of Nessler's reagent is as follows: 0, 50, 150, 250, 350, 450, and 500 μL of 10 mg/L NH_4Cl standard solution was transferred into seven colorimetric tubes respectively. And, the samples tubes were diluted with ultrapure water to 5 mL. Then, 100 μL potassium sodium tartrate was added to the sample tubes. After mixing well, 150 μL of Nessler's reagent was also added to the sample tubes and mixed for ageing 20 min, and then measured by the SHIMADZU UV-3600 spectrometer. The absorbance at 420 nm was measured and plotted as a function of ammonia concentration.

Computational methods

DFT calculations were performed by the Vienna ab initio simulation Package (VASP),[S14] using a planewave basis set with energy cutoff of 500 eV, projector augmented wave (PAW) pseudopotentials,[S15] and the generalized gradient approximation parameterized by Perdew, Burke, and Ernzerhof (GGA-PBE) for the exchange-correlation functional.[S16] The Grimme's DFT-D3 scheme for dispersion correction was adopted for better description of the interactions between the gold clusters and reaction intermediates involved in N_2 fixation.[S17] The clusters were placed in a cubic supercell with a dimension of 30 Å, and its

Brillouin zone was sampled by the Γ point. All of structures were fully optimized for the ionic and electronic degrees of freedom using the convergence criteria of 10^{-4} eV for electronic energy and 10^{-2} eV/Å for the forces on each atom. The kinetic barriers and transition states for elementary steps of N_2 fixation were calculated by the climbing-image nudged elastic band (CI-NEB) method implemented in VASP,[S18] with five images to mimic the reaction path. The intermediate images were relaxed until the perpendicular forces were less than 10^{-2} eV/Å. To simplify our models and improve the computational efficiency, the -triphenylphosphine (-PPh_3) and -phenylethanethiol (-SC_2H_4Ph) ligands were replaced by -trimethylphosphine -$P(CH_3)_3$ and -SCH_3, respectively, which, according to our test calculations, does not affect the binding strength of the cluster with N_2 molecule.

We used a slab model consisting of the six atomic layers of anatase TiO_2(101) and 2 × 3 unit cells for the lateral dimensions, including the 32 Ti atoms and 64 O atoms (Figure. S2). A single oxygen atom of surface and subsurface was removed to simulate oxygen vacancy on the surface (TiO_2-Ov_1) and subsurface (TiO_2-Ov_2) of anatase TiO_2(101). For model of ligand-protected Au_4Ru_2 cluster, the -triphenylphosphine (-PPh_3) and -phenylethanethiol (-SC_2H_4Ph) ligands were replaced by -trimethylphosphine -$P(CH_3)_3$ and -SCH_3, respectively, which, according to our test calculations, does not affect the binding strength of the cluster with N_2 molecule (Figure S12). We examined the adsorption of a N_2 molecule on the cluster with one ligand shed from either Ru or Au atom (Figure S14 and Table S1). Our calculations show that the exposed Au atom cannot chemisorb N_2 molecule. Only the exposed Ru atom in the cluster has activity for N_2 fixation.

To characterize the interactions between ligand-protected gold clusters and N_2 molecule, we defined the adsorption energy as

$$\Delta E_{*N2} = E_{total} - E_{cluster} - E_{N2} \quad (1)$$

where E_{total} and $E_{cluster}$ are the energies of a gold cluster with and without adsorption of a N_2 molecule, respectively; E_{N2} is the energy of the N_2 molecule in the gas phase.

Supporting Figures

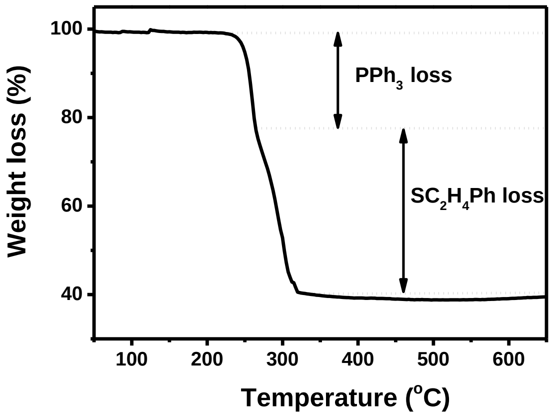

Figure S1. TGA curve of the $Au_4Ru_2(PPh_3)_2(SC_2H_4Ph)_8$ cluster.

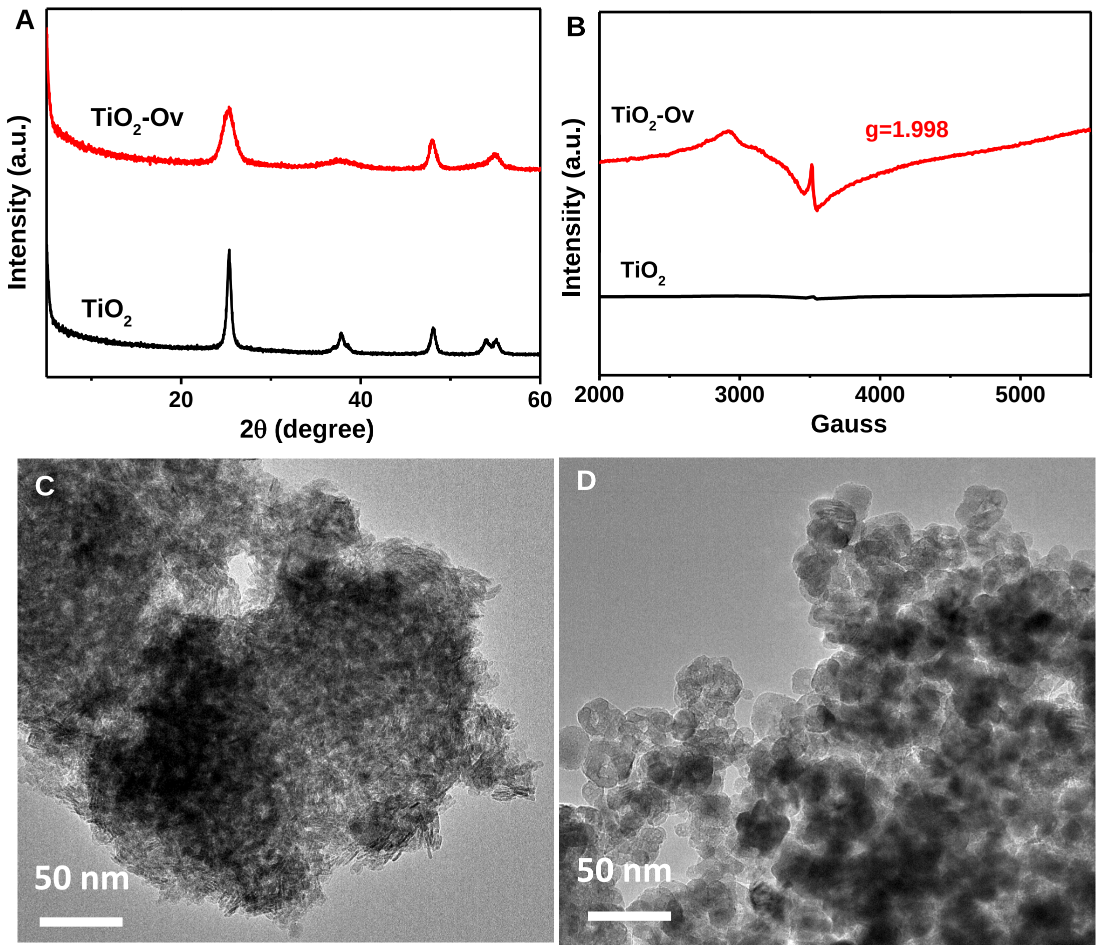

Figure S2. (A) XRD patterns and (B) EPR spectra of TiO_2-Ov and TiO_2. TEM images of (C) TiO_2-Ov and (D) TiO_2.

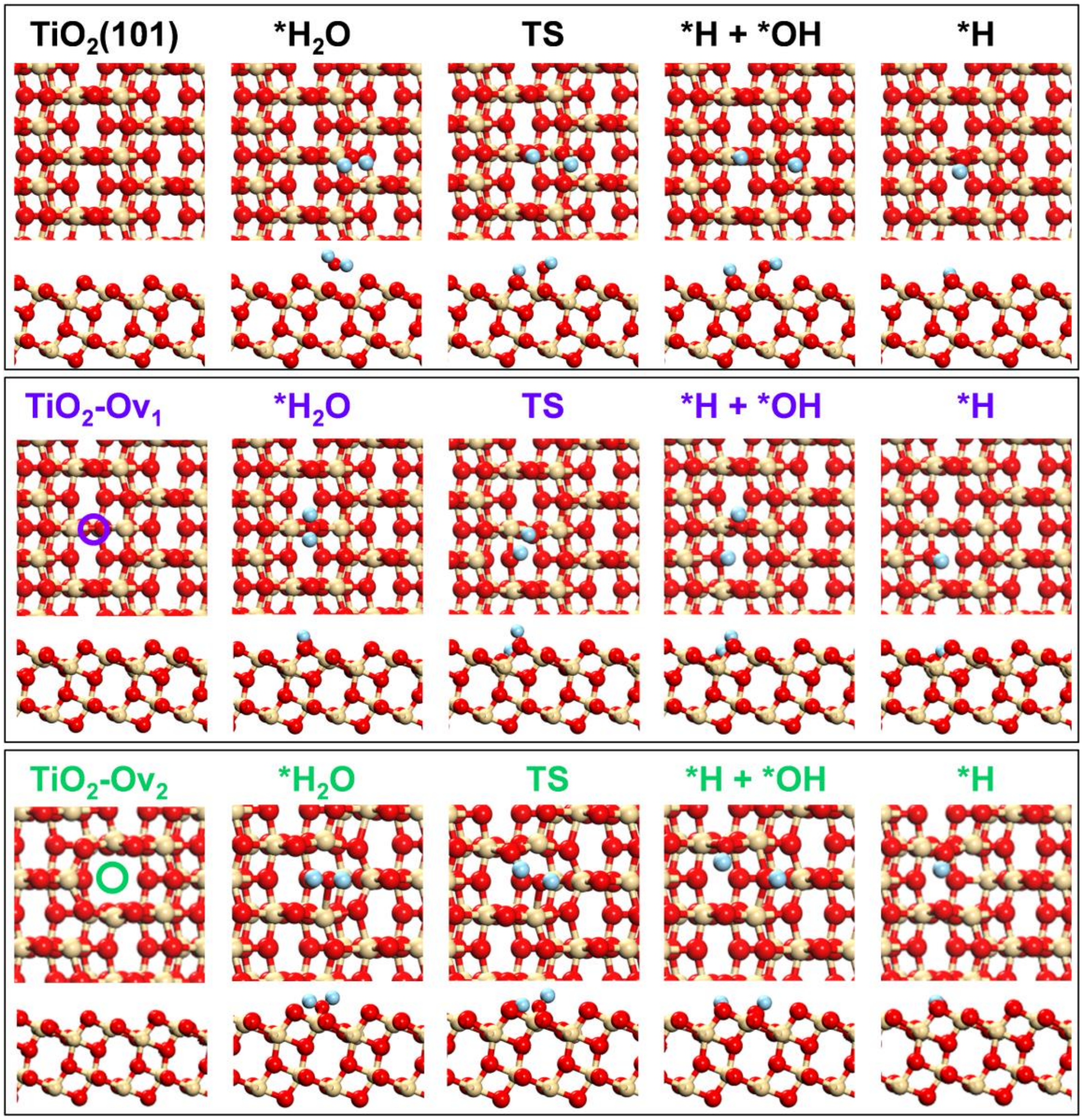

Figure S3. Structures of water adsorption and dissociation into *H and *OH species adsorbed on the anatase TiO_2(101) surface without defect (TiO_2) and with an oxygen vacancy on the surface (TiO_2-Ov_1) and subsurface (TiO_2-Ov_2). The H, O and Ti atoms are shown in light blue, red and yellowish colors, respectively.

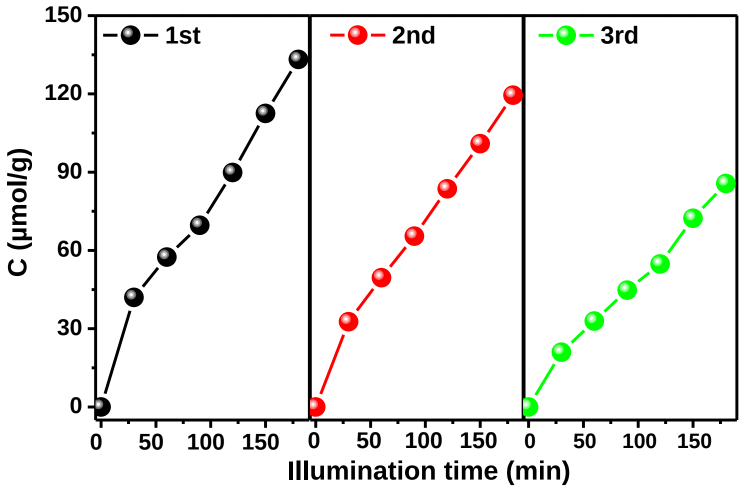

Figure S4. Recyclability of the Au_4Ru_2/TiO_2-Ov catalyst for photocatalytic N_2 reduction.

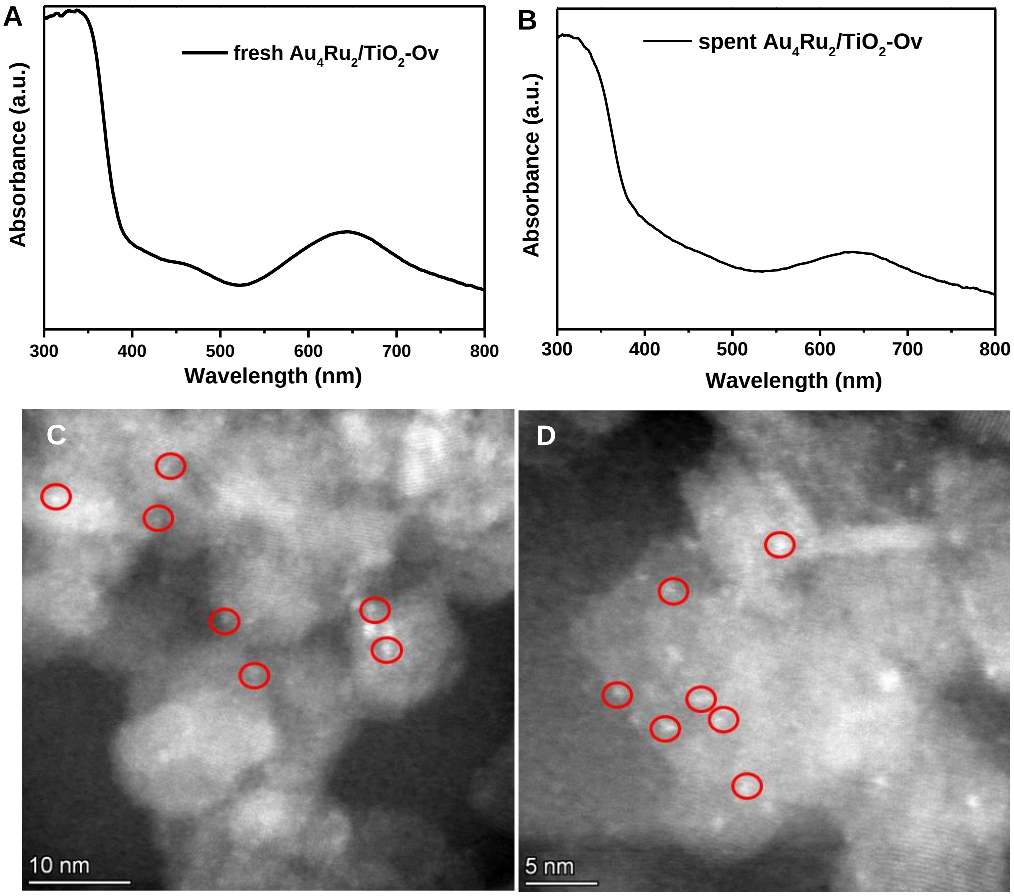

Figure S5. The diffuse reflectance optical spectra of (A) the fresh and (B) the spent Au_4Ru_2/TiO_2-Ov. HRTEM images of (C) the fresh and (D) the spent Au_4Ru_2/TiO_2-Ov.

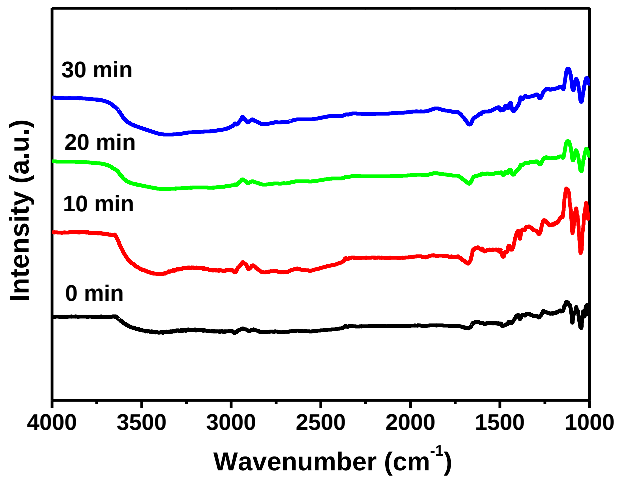

Figure S6. In situ FTIR spectra of the photocatalytic N_2 reduction over Au_4Ru_2/TiO_2-Ov under full spectrum illumination in the absence water.

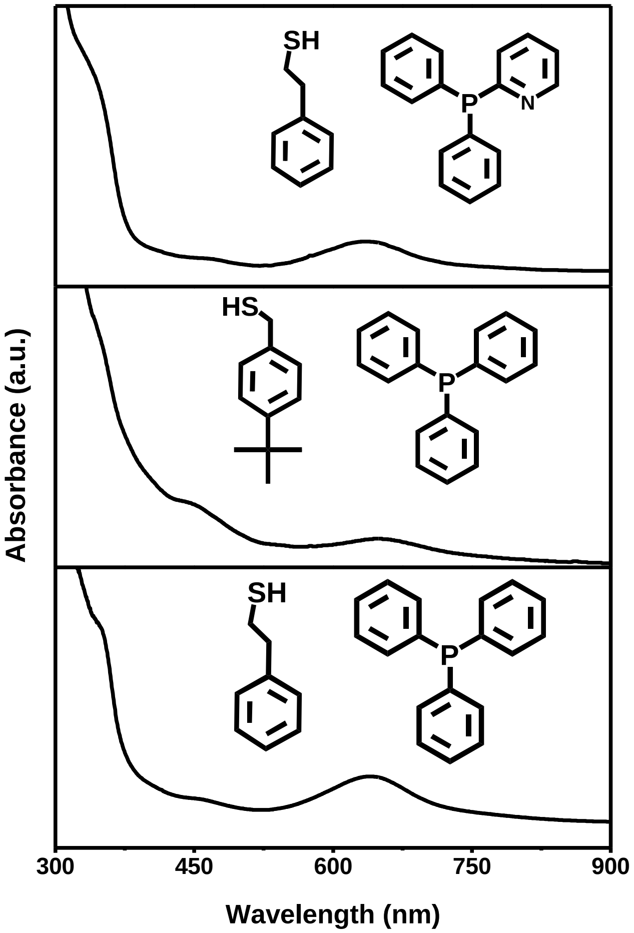

Figure S7. UV-vis spectra of the Au_4Ru_2 clusters protected by different ligands.

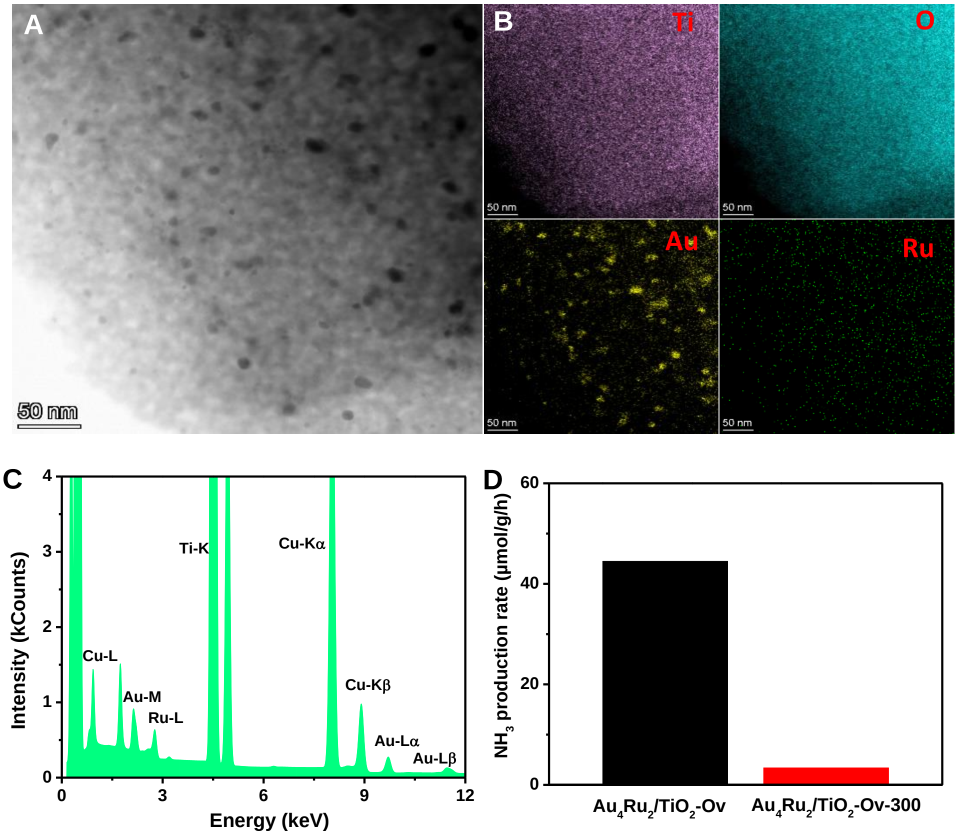

Figure S8. (A) TEM image, (B) Element composition analysis, and (C) EDS elemental profiles of Au_4Ru_2/TiO_2-Ov-300 (Au_4Ru_2/TiO_2-Ov-300 was obtained by Au_4Ru_2/TiO_2-Ov calcined at 300 °C in air for 2 h). (D) The photocatalytic activity of the Au_4Ru_2/TiO_2-Ov and Au_4Ru_2/TiO_2-Ov-300 for N_2 reduction.

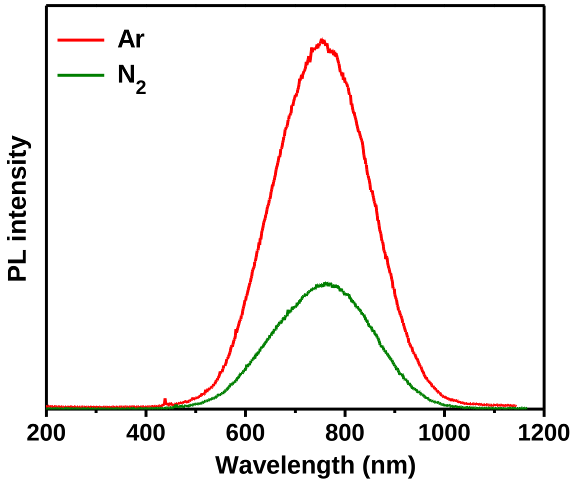

Figure S9. Room-temperature steady-state PL spectra of Au_4Ru_2/TiO_2-Ov under Ar and N_2 atmospheres, respectively.

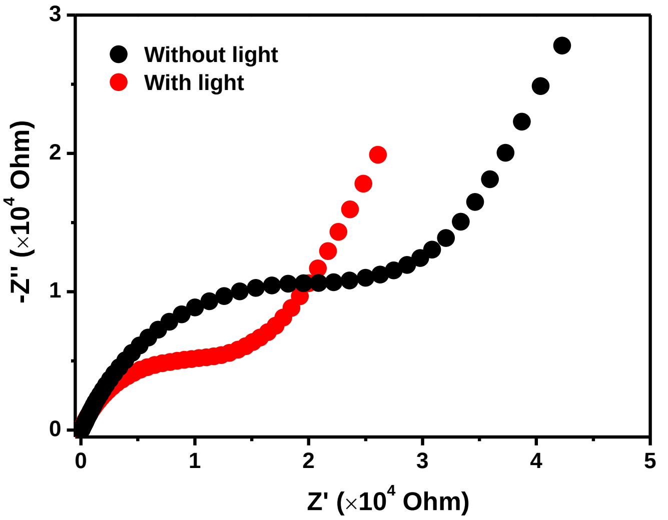

Figure S10. Electrochemical impedance spectra of Au_4Ru_2/TiO_2-Ov with and without light (300W Xenon lamp).

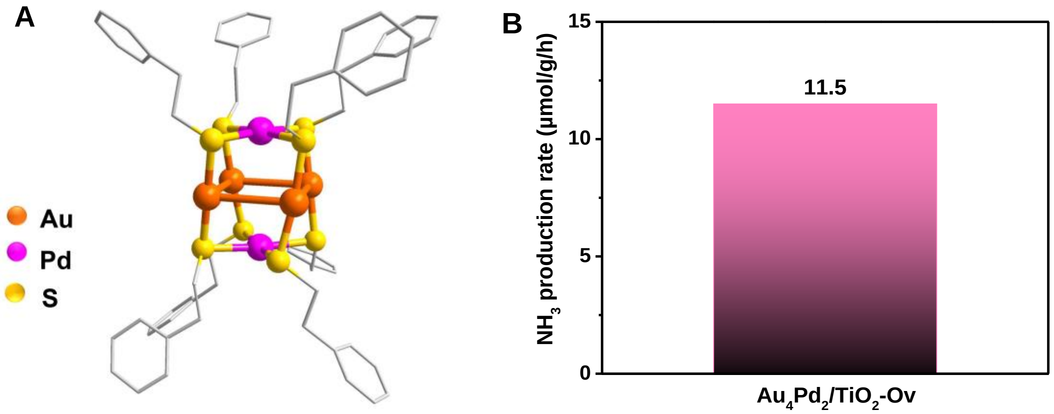

Figure S11. (A) The atomic structure of $Au_4Pd_2(SC_2H_4Ph)_8$. H atoms are omitted for clarity. (B) The photocatalytic activity of the Au_4Pd_2/TiO_2-Ov for N_2 reduction.

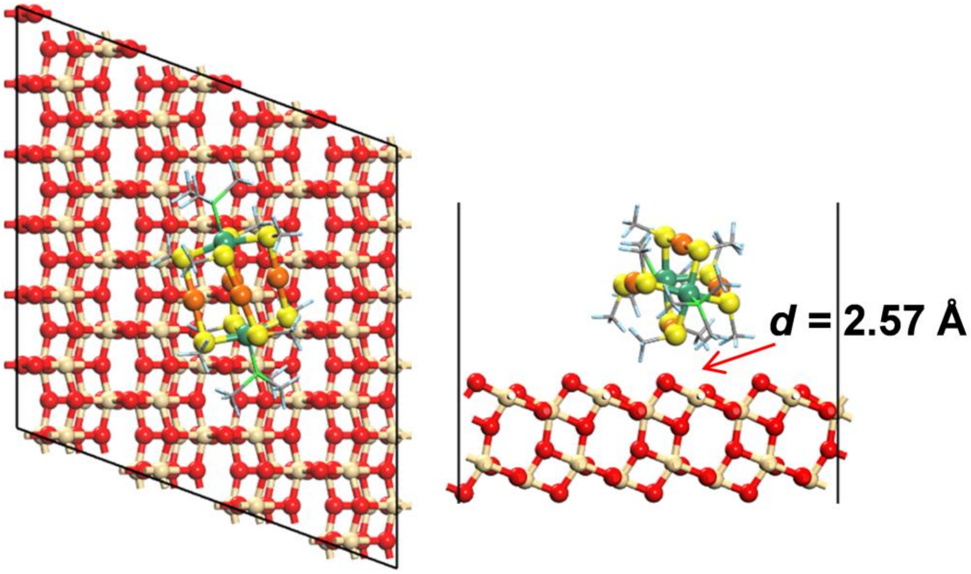

Figure S12. Top (lef panel) and side views (right panel) of optimized Au_4Ru_2 on the surface of anatase TiO_2(101). The black box indicates the lateral dimension of the supercell used for DFT calculations. The H, C, O, P, S, Au, Ru and Ti atoms are shown in light blue, gray, red, green, yellow, orange, blackish green and yellowish, respectively. The closest distance between the cluster and substrate is shown as *d*.

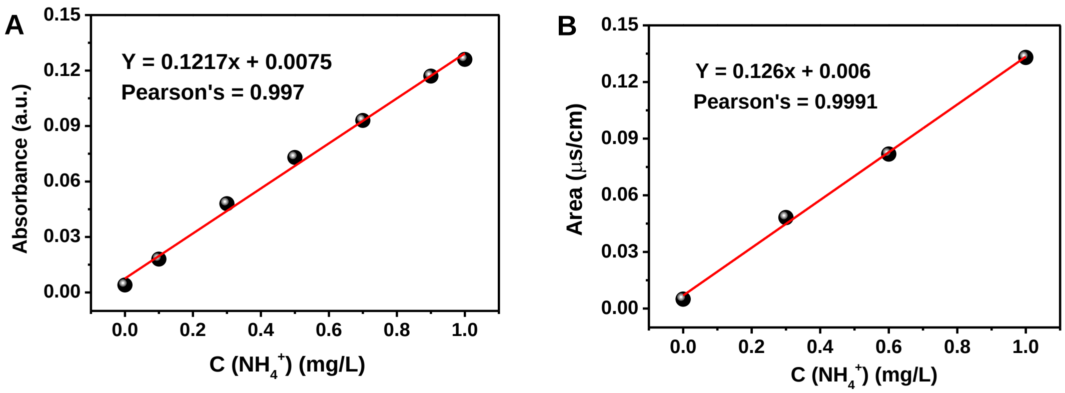

Figure S13. Standard curves for NH_4^+ with (A) Nessler's reagent and (B) ion chromatography.

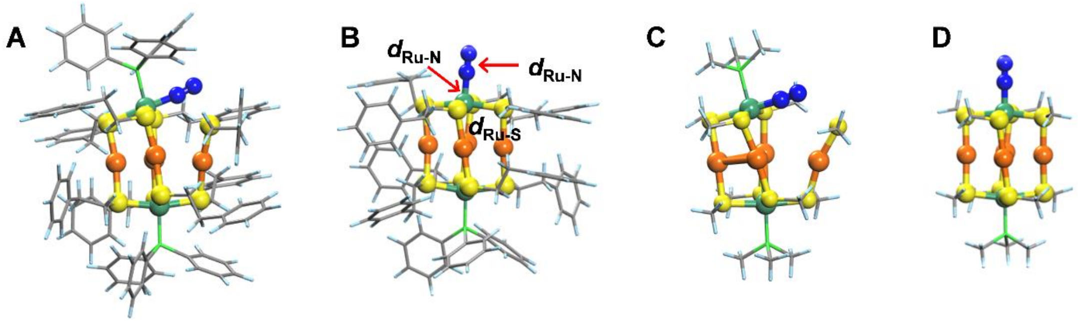

Figure S14. Structures of N_2 adsorption on (A) $Au_4Ru_2(SC_2H_4Ph)_8(PPh_3)_2$, (B) $Au_4Ru_2(SC_2H_4Ph)_8PPh_3$, (C) $Au_4Ru_2(SCH_3)_8(P(CH_3)_3)_2$ and (D) $Au_4Ru_2(SCH_3)_8P(CH_3)_3$. The H, C, N, S, Au and Ru are shown in light blue, gray, blue, yellow, orange and green colors, respectively. When the cluster is fully covered by ligands (A, C), N_2 adsorption leads to severe structure distortion.

Table S1. Geometrical parameters including the bond length of N–N ($d_{N–N}$), Ru–N ($d_{Ru–N}$) and Ru–S ($d_{Ru–S}$) bonds and N_2 adsorption energy (ΔE_{*N2}) for a N_2 molecule on the ligand-protected Au_4Ru_2 clusters shown in Figure S14.

	$Au_4Ru_2(SC_2H_4Ph)_8(PPh_3)_2$	$Au_4Ru_2(SC_2H_4Ph)_8PPh_3$	$Au_4Ru_2(SCH_3)_8(P(CH_3)_3)_2$	$Au_4Ru_2(SCH_3)_8P(CH_3)_3$
d_{N-N} (Å)	1.13	1.14	1.13	1.14
d_{Ru-N} (Å)	1.90	1.84	1.90	1.84
d_{Ru-S} (Å)	4.18	2.27	4.83	2.27
ΔE_{*N2} (eV)	–1.19	–1.25	–1.28	–1.33

Supporting References

(S1). S. Yamazoe, S. Matsuo, S. Muramatsu, S. Takano, K. Nitta and T. Tsukuda, *Inorg. Chem.* 2017, **56**, 8319-8325.

(S2). T. Huang, L. Huang, Y. Jiang, F. Hu, Z. Sun, G. Pan and S. Wei, *Dalton Trans.* 2017, **46**, 12239-12244.

(S3). L. C. McKenzie, T. O. Zaikova and J. E. Hutchison, *J. Am. Chem. Soc.* 2014, **136**, 13426-13435.

(S4). A. Das, C. Liu, H. Y. Byun, K. Nobusada, S. Zhao, N. Rosi and R. Jin, *Angew. Chem. Int. Ed.* 2015, **54**, 3140-3144.

(S5). S.Wang, H. Abroshan, C. Liu, T. Luo, M. Zhu, H. J. Kim, N. L. Rosi and R. Jin, *Nat Commun.* 2017, **8**, 848.

(S6). Y. Zhu, H. Qian, B. Drake and R. Jin, *Angew. Chem. Int. Ed.* 2010, **49**, 1295-1298.

(S7). C. Zeng, Y. Chen, K. Iida, K. Nobusada, K. Kirschbaum, K. L. Lambright and R. Jin, *J. Am. Chem. Soc.* 2016, **138**, 3950-3953.

(S8). C. Zeng, Y. Chen, G. Li and R. Jin, *Chem. Commun.* 2014, **50**, 55-57.

(S9). A. Das, T. Li, K. Nobusada, C. Zeng, N. L. Rosi and R. Jin, *J. Am. Chem. Soc.* 2013, **135**, 18264-18267.

(S10). J. Chen, L. Liu, X. Liu, L. Liao, S. Zhuang, S. Zhou, J. Yang and Z. Wu, *Chem. Eur. J.* 2017, **23**, 18187-18192.

(S11). Y. Zhao, Y. Zhao, R. Shi, B. Wang, G. I. N. Waterhouse, L. Wu, C. Tung and T. Zhang, *Adv. Mater.* 2019, 1806482.

(S12). H. Li, J. Shang, Z. Ai and L. Zhang, *J. Am. Chem. Soc.* 2015, **137**, 6393-6399.

(S13). W. G. Watt, and J. D. Chrisp, *Anal. Chem.* 1952, **24**, 2006-2008.

(S14). G. Kresse, and J. Furthmüller, *Phys. Rev. B* 1996, **54**, 11169-11186.

(S15). G. Kresse, and D. Joubert, *Phys. Rev. B* 1999, **59**, 1758-1775.

(S16). J. P. Perdew, K. Burke and M. Ernzerhof, *Phys. Rev. Lett.* 1996, **77**, 3865-3868.

(S17). S. Grimme, J. Antony, S. Ehrlich and H. Krieg, *J. Chem. Phys.* 2010, **132**, 154104-154123.

(S18). G. Henkelman, B. P. U. Beruaga and H. Jónsson, *J. Chem. Phys.* 2000, **113**, 9901-9904.
